# The IBD-FITT Study—Moderate-Intensity Exercise for Patients with Active Inflammatory Bowel Disease: An Open-Label Randomized Controlled Trial

**DOI:** 10.3390/jcm15062106

**Published:** 2026-03-10

**Authors:** Ken Lund, Torben Knudsen, Jens Kjeldsen, Kate Lykke Lambertsen, Rasmus Gaardskær Nielsen, Carsten Bogh Juhl, Bente Mertz Nørgård

**Affiliations:** 1 Center for Clinical Epidemiology, Odense University Hospital, 5000 Odense C, Denmark; 2 Research Unit of Clinical Epidemiology, Department of Clinical Research, University of Southern Denmark, 5000 Odense C, Denmark; 3 Department of Medical Gastroenterology, Hospital of Southwest Jutland, 6700 Esbjerg, Denmark; 4 Department of Regional Health Science, Center Southwest Jutland, University of Southern Denmark, 6700 Esbjerg, Denmark; 5 Department of Medical Gastrointestinal Diseases, Odense University Hospital, 5000 Odense C, Denmark; 6 Research Unit of Medical Gastroenterology, Department of Clinical Research, University of Southern Denmark, 5000 Odense C, Denmark; 7 Department of Neurology, Odense University Hospital, 5000 Odense C, Denmark; 8 Department of Neurobiology Research, Institute of Molecular Medicine, University of Southern Denmark, 5230 Odense M, Denmark; 9 BRIDGE–Brain Research–Inter Disciplinary Guided Excellence, Department of Clinical Research, University of Southern Denmark, Campusvej 55, 5230 Odense M, Denmark; 10 Hans Christian Andersen Children’s Hospital, Odense University Hospital, 5000 Odense C, Denmark; 11 Research Unit of Pediatrics, Department of Clinical Research, University of Southern Denmark, 5230 Odense M, Denmark; 12 Department of Sports Science and Clinical Biomechanics, University of Southern Denmark, 5230 Odense M, Denmark; 13 Department of Physiotherapy and Occupational Therapy, Herlev and Gentofte Hospital, Copenhagen University Hospital, 2730 Herlev, Denmark

**Keywords:** inflammatory bowel disease, Crohn’s disease, ulcerative colitis, exercise, randomized controlled trial

## Abstract

**Background:** Exercise has been suggested as a supplementary modality for Inflammatory Bowel Disease (IBD), but supporting evidence remains scarce. We aimed to assess whether a 12-week physical exercise intervention improves quality of life (QOL) in adults with active IBD. **Methods:** An open-labeled randomized controlled trial examining the efficacy of a 12-week physical exercise intervention on QOL in adults (18–65 years) with active IBD. Participants were randomized 1:1 into either an intervention group, with two weekly supervised exercise sessions and one home session, or a control group with standard care. QOL by the Inflammatory Bowel Disease Questionnaire (IBDQ) was the primary outcome. Secondary outcomes were the European Quality of Life 5 Dimensions (EQ5D), waist circumference, blood pressure, disease activity, and lipid status. Explorative outcomes were C-reactive protein, fecal calprotectin, and cytokines (interleukin-6, -8, and -10 and tumor necrosis factor). **Results:** We screened 183 patients and included 44 participants, with 22 in each group. Eleven participants completed more than 50% of the exercise sessions. Among the participants, 17 were male, 27 were female, and the mean age was 37 years. The mean IBDQ scores at week 12 showed no statistically significant difference: 172 for the intervention group (95%CI: 158–185) and 164 for the control group (95%CI: 151–178). No clinically significant differences for secondary or exploratory outcomes were found. **Conclusions**: We did not find any difference in the QOL after a 12-week exercise intervention in patients with active IBD compared to standard care. Recruiting proved difficult, as did adherence to exercise sessions, mostly due to scheduling issues.

## 1. Introduction

Inflammatory Bowel Diseases (IBD), Crohn’s disease (CD), and ulcerative colitis (UC) are chronic autoimmune diseases characterized by fluctuating disease activity. Complex medical therapies and surgical interventions are used to manage inflammation [1,2]. Disease control is important for maintaining quality of life (QOL), physical function, and study/work participation in an increasing population of IBD [3].

Structured physical exercise has been suggested as a supplementary treatment modality [4,5]. Exercise can potentially improve the immunological response, psychological health, and muscle and bone strength [5,6]. Furthermore, exercise may have a beneficial influence on patients’ well-being and QOL [4,5,7]. Although the mechanism is not fully understood, exercise increases cytokines, improving the regulation of inflammation and promoting an anti-inflammatory state that may reduce the disease activity in IBD [4,5]. However, evidence of its efficacy is limited, with a few empirical studies, mostly among patients in remission, which may have limited the measurable effect of exercise on inflammatory markers [4,5,6,7].

In adult patients with active IBD, we aimed to examine the efficacy of a 12-week physical exercise intervention on QOL, clinical disease activity, inflammatory markers, and immune response. For additional information, see the previously described protocol [8].

## 2. Materials and Methods

### 2.1. Study Design and Setting

This is an open-label randomized trial with two parallel groups randomized 1:1 in blocks of 4 after eligibility screening as previously described [8]. The intervention group received two supervised exercise sessions and one home session weekly, and the control group received standard care over 12 weeks. The study took place in gastroenterology outpatient clinics at two separate hospitals: The Department of Medical Gastrointestinal Diseases at Odense University Hospital and The Department of Medicine at Southwest Jutland Hospital. The intervention took place at an authorized physiotherapy clinic located in Odense and at the Department of Physiotherapy at Southwest Jutland Hospital.

### 2.2. Participants and Interventions

We included adults (18–65 years) with active IBD, indicated by a score of >5 points on the Harvey Bradshaw Index (HBI) for CD or on the Simple Clinical Colitis Activity Index (SCCAI) for UC, or a fecal calprotectin higher than 200 mg/g or C-reactive protein equal to or higher than 6 mg/L. Inclusion and exclusion criteria are detailed in the published protocol [8]. A 12-week moderate exercise program was designed to be accessible and adaptable regardless of prior exercise state [8]. Examples of exercises include biking, squats, and stepping. The intervention included two 60 min supervised exercise sessions per week and one home exercise session. A moderate exercise level was defined as 60–80% of maximum heart rate, and exercises were individually tailored by a physiotherapist, as described previously [8]. Adverse events were monitored during the exercise sessions [8]. Data were captured in paper diaries, entered into a database by a student worker, and controlled by the first author [8].

### 2.3. Outcomes

The primary outcome was QOL using the Inflammatory Bowel Disease Questionnaire (IBDQ) [8]. Secondary outcomes were European Quality of Life–5 Dimensions (EQ5D), body mass index, waist circumference, blood pressure, disease activity scores, lipid status, and explorative biomarkers for inflammation [8]. We analyzed the exploratory outcomes (cytokines) using the V-PLEX proinflammatory panel from Mesoscale Diagnostics (MSD, Rockville, MA, USA) on a SECTOR Imager 6000 Plate Reader (Mesoscale Discovery) according to the manufacturer’s instructions.

### 2.4. Statistics

Descriptively, categorical variables were presented using frequency, counts, median, and interquartile range (IQR). For continuous data, means with standard deviation (SD) were reported. Comparative analyses were conducted using an intention-to-treat approach, under the assumption of missing at random (MAR). To assess the validity of MAR, the baseline for participants who completed the study was compared to that of participants with missing outcome data. Multilevel mixed-effect linear and logistic regression models were employed for group comparisons, adjusting for age, sex, and type of disease as covariates when feasible, reporting means and 95% confidence intervals (95%CI). Additionally, a per-protocol subgroup analysis was performed to evaluate the change in VO_2_max (according to a Watt-max cycling test as previously described) [8] among participants who completed more than 50% of the sessions.

### 2.5. Ethical Approval

This study was approved by the Regional Ethics Committee of the Region of Southern Denmark (protocol code: S-20200003) on 27 February 2020 and registered at Clinicaltrials.gov (NCT04816812). This study was conducted in accordance with the Declaration of Helsinki. Informed consent was obtained from all subjects involved in the study.

## 3. Results

**Figure 1:**
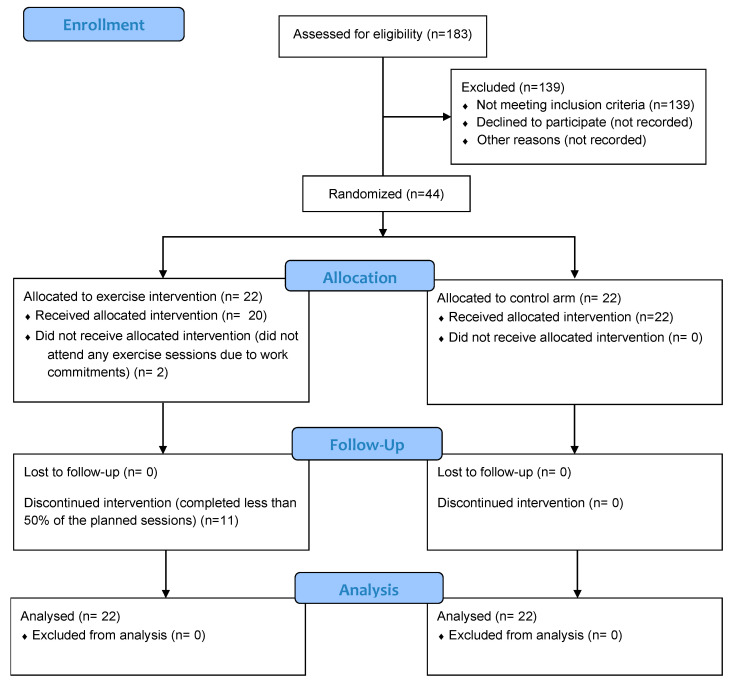
Consort flow diagram.

We screened 183 patients and included 44 participants, 22 in the intervention group and 22 in the control group. In the intervention group, 20 of 22 participants attended exercise sessions: 3 completed over 80% of supervised sessions, 11 over 50%, and 7 completed over 80% of home sessions. Recruitment and adherence to the exercise intervention were hindered by the time the exercise sessions were scheduled, which often conflicted with the participants’ work commitments. Figure 1 shows the consort flow diagram. Table 1 shows descriptive characteristics.

The IBDQ score showed no significant difference of 8 points between the intervention and control groups, with predicted mean scores of 172 (95%CI: 158–185) and 164 (95%CI: 151–178) at week 12. Five did not answer the IBDQ questionnaire completely at week 0 and week 12. Twelve had missing answers to the IBDQ questionnaire at week 0 or 12. Those who did not respond were not statistically significantly different in terms of age, sex, or type of disease. The EQ5D was divided into five subdomains plus the visual rating scale; none were statistically significantly different, except for the domain of pain and discomfort, which was significantly lower in the intervention group at week 12. No statistically significant differences were found in waist circumference and systolic blood pressure. Diastolic blood pressure and body mass index were statistically significantly lower and higher for the intervention group, respectively. For the clinical disease score, HBI, and SCCAI, a non-significant reduction in the score was observed at week 12. Lipid status and hemoglobin showed no significant difference and were generally within the normal range. Albumin was omitted due to missing data (only three measurements). No significant differences were found for explorative outcomes. Table 2 shows outcome measures.

In a per-protocol sub-analysis, participants who completed more than 50% showed a non-significant change in the relative VO_2_max (mL/kg/min) based on a bike Watt-max test from 30.25 (±SD: 4.41) to 31.60 (±SD: 3.54) at week 12. The mean of perceived exertion on the BORG scale [9] for all completed sessions was 12.5 points (±SD: 1.25). No adverse events were registered.

## 4. Discussion

In this randomized trial, we examined the efficacy of a 12-week supervised exercise program for patients with active IBD. We did not find a statistical difference between groups in the primary outcome of QOL or secondary outcomes, including disease activity and exploratory outcomes for inflammatory markers and immune response.

Structured exercise has been suggested for improving QOL and other outcomes in patients with IBD [4,5,7]. Still, evidence from randomized studies is limited, with varying results regarding efficacy, ranging from no changes to improvement for various outcomes based on various interventions [4,5,6,7]. Previously, improvement in QOL after exercise interventions has been shown [4], even though the studies are heterogeneous. We did not observe a significant difference in the QOL score, consistent with other studies, despite their use of different interventions and inclusion of patients in remission [4,6]. A recent systematic review including a meta-analysis has demonstrated a significant reduction in disease activity in exercise groups versus controls, but no significant changes for IBDQ scores or inflammatory markers [4]. The systematic review demonstrated the heterogeneity of the studies in changes to the IBDQ scores, with a mean difference ranging from 17.5 to −8.3 [4]. This variation may reflect differences in study populations, intervention types, and duration. Tew et al. [10] report a decline in IBDQ scores in their pilot randomized controlled trial of 36 adult patients with Crohn’s disease who undertook high-intensity and moderate-intensity continuous training over 6 months. Jones et al. [11] observed a mean of 4 points in the IBDQ score among 47 adults with stable Crohn’s disease following a 6-month intervention. Seeger et al. [6] observed a modest mean increase of 1 point in the short IBDQ score in their randomized trial of 45 patients with Crohn’s disease completing endurance and muscle training. These findings align with ours, showing no significant difference in the IBDQ score, even though our study has a shorter evaluation period of 3 months, differences in intervention types, and lower participant adherence. There are several important factors to consider when interpreting our results. Our study had a small sample size and low adherence to exercise sessions. The limited recruitment and adherence were hindered by several factors, even though regular screening was done. One factor could be that we were targeting participants with active disease, and another could be the scheduled time of day for the supervised exercise session or the length of 12 weeks. The exercise sessions were only offered at one morning hour (7–8 am) in Odense or during the daytime (between 9 am and 3 pm) in Esbjerg, which often conflicted with work commitments for most participants. Patients with IBD may also experience other barriers to participating in exercise, alone or in combination, such as fatigue, fear of increased toilet urgency, and abdominal pain [12].

Our study has several limitations. Recruitment was from November 2021 to May 2023, during which the coronavirus pandemic advanced in Denmark. To overcome this and to address the low number of participants, the inclusion period was extended by 6 months to approach our target of 150 participants [8]. Early in the project, the original inclusion criteria were revised to require either a disease score above 5 (HBI or SCCAI) or an increased clinical disease marker [8]. When assessing QOL scores, the minimal clinically important difference is important, and changes of 16 points in the IBDQ score are relevant [13], though the difference here was smaller. The treatment of active IBD may also contribute to the improvement in QOL in both groups. The open-label design in combination with self-reported QOL could be prone to an expectancy bias, which should be taken into consideration for these results. The limited adherence to the exercise intervention should be acknowledged, as this may limit adequate assessment of the efficacy. Lastly, some significant baseline differences, BMI and fecal calprotectin, may have influenced the results. Improving QOL in patients in remission remains a key focus for future studies.

Despite the limitations, some strengths are apparent. We used a randomized design to examine the efficacy of an exercise intervention in participants with active IBD, focusing on QOL and changes in inflammatory markers and cytokines. However, recruiting participants and ensuring adherence to the exercise sessions turned out to be challenging in our setup. Focusing on patients in remission and offering flexible scheduling outside working hours might have increased recruitment and adherence. Among the participants who completed the exercise sessions, the overall tolerance was good.

## 5. Conclusions

We did not observe a statistically significant difference in QOL after 12 weeks of exercise sessions in patients with active IBD compared to a control group. The recruitment and adherence to exercise sessions were low, mostly hindered by the time of day for the exercise sessions conflicting with work commitments. The number of participants was limited, reducing the ability to truly assess the efficacy of exercise in patients with active IBD. The results must be interpreted with this in mind, and the time of day for an exercise session is important for future studies. Physical activity may play an important role for patients with IBD in remission, offering numerous benefits, from cardiovascular health to better mental health, or potentially helping to prevent future relapse, which may be explored further in future studies. 

## Figures and Tables

**Table 1:** Descriptive characteristics of participants in the IBD-FITT study.

Characteristics	Intervention	Control
	n (%)	n (%)
Total (N)	22	22
Age at inclusion		
Median years (IQR)	37 (26–47)	38 (29–43)
Sex		
Male	11 (50%)	6 (27%)
Female	11 (50%)	16 (73%)
Type of IBD		
Ulcerative colitis (UC)	9 (41%)	5 (23%)
Crohn’s disease (CD)	13 (59%)	17 (77%)
Medications for UC or CD		
Biologics	19 (86%)	20 (91%)
Immunosuppressants	5 (23%)	2 (9%)
5-aminosalicylates	5 (23%)	2 (9%)
Other	0 (0%)	2 (9%)
Body mass, mean (SD)		
Body mass index (kg/m^2^)	31 (6)	27 (5)
Disease activity at inclusion, mean (SD)		
Fecal calprotectin mg/g	198 (393)	761 (999)
C-reactive protein mg/L	7 (7)	6 (7)
Trial Location		
Odense University Hospital	16 (73%)	7 (32%)
Hospital of Southwest Jutland	6 (27%)	15 (68%)

**Table 2:** Main, secondary, and explorative outcome measures for the intervention group and the control group.

			Intervention Group	Control Group
Main outcome				
Inflammatory Bowel Disease Questionnaire (IBDQ) score				
	Week 0, mean (±SD)		151 (30)	153 (30)
	Week 12, mean (±SD)		171 (35)	164 (28)
Secondary outcomes				
European Quality of Life–5 dimensions (EQ5D, not disease-specific)				
EQ5D—Mobility				
	Week 0, n (%)			
		No problems, score 1	7 (39%)	10 (59%)
		Any problems, score ≥ 2	11 (61%)	7 (41%)
	Week 12, n (%)			
		No problems, score 1	8 (53%)	7 (47%)
		Any problems, score ≥ 2	7 (47%)	8 (53%)
EQ5D—Self-care				
	Week 0, n (%)			
		No problems, score 1	7 (39%)	2 (12%)
		Any problems, score ≥ 2	11 (61%)	15 (88%)
	Week 12, n (%)			
		No problems, score 1	6 (40%)	1 (7%)
		Any problems, score ≥ 2	9 (60%)	14 (93%)
EQ5D—Standard activities				
	Week 0, n (%)			
		No problems, score 1	11 (61%)	8 (47%)
		Any problems, score ≥ 2	7 (39%)	9 (53%)
	Week 12, n (%)			
		No problems, score 1	10 (67%)	10 (67%)
		Any problems, score ≥ 2	5 (33%)	5 (33%)
EQ5D—Pain/discomfort				
	Week 0, n (%)			
		No problems, score 1	16 (89%)	17 (100%)
		Any problems, score ≥ 2	2 (11%)	0 (0%)
	Week 12, n (%)			
		No problems, score 1	14 (93%)	14 (93%)
		Any problems, score ≥ 2	1 (7%)	1 (7%)
EQ5D—Anxiety/depression				
	Week 0, n (%)			
		No problems, score 1	11 (61%)	14 (82%)
		Any problems, score ≥ 2	7 (39%)	3 (18%)
	Week 12, n (%)			
		No problems, score 1	12 (80%)	11 (73%)
		Any problems, score ≥ 2	3 (20%)	4 (27%)
EQ5D—EQ VAS				
	Week 0, mean (±SD)		64 (22)	58 (22)
	Week 12, mean (±SD)		71 (22)	63 (21)
Waist circumferences (cm)				
	Week 0, mean (±SD)		102 (18)	95 (14)
	Week 12, mean (±SD)		102 (15)	94 (15)
Body mass index (kg/m^2^)				
	Week 0, mean (±SD)		31 (6)	27 (5)
	Week 12, mean (±SD)		30 (5)	27 (6)
Blood pressure				
	Week 0			
		Systolic, mean (±SD)	125 (11)	123 (13)
		Diastolic, mean (±SD)	81 (9)	81 (13)
	Week 12			
		Systolic, mean (±SD)	124 (10)	129 (15)
		Diastolic, mean (±SD)	75 (7)	84 (9)
Clinical disease activity				
Harvey Bradshaw Index				
	Week 0, mean (±SD)		6 (5)	4 (4)
	Week 12, mean (±SD)		3 (3)	3 (3)
Simple Clinical Colitis Activity Index				
	Week 0, mean (±SD)		2 (2)	4 (4)
	Week 12, mean (±SD)		2 (3)	3 (4)
Lipid status				
Low-density lipoprotein (LDL) mmol/L				
	Week 0, mean (±SD)		2.5 (0.8)	2.6 (0.8)
	Week 12, mean (±SD)		2.5 (0.9)	2.7 (0.9)
High-density lipoprotein (HDL) mmol/L				
	Week 0, mean (±SD)		1.3 (0.4)	1.3 (0.3)
	Week 12, mean (±SD)		1.4 (0.5)	1.3 (0.3)
Triglycerides mmol/L				
	Week 0, mean (±SD)		2.1 (1.6)	1.5 (0.6)
	Week 12, mean (±SD)		1.5 (0.8)	1.4 (0.6)
Hemoglobin A (Hbac1), mmol/mol				
	Week 0, mean (±SD)		35.9 (6.9)	34.2 (2.7)
	Week 12, mean (±SD)		35.1 (5.5)	34.1 (2.7)
Albumin mmol/mol				
	Week 0, mean (±SD)		Omitted due to many missing data	Omitted due to many missing data
	Week 12, mean (±SD)		Omitted due to many missing data	Omitted due to many missing data
Explorative outcomes				
Fecal calprotectin, µg/g				
	Week 0, mean (±SD)		185.1 (199.7)	552.9 (931.6)
	Week 12, mean (±SD)		133.8 (143.6)	292.3 (437.5)
C-reactive protein, mg/L				
	Week 0, mean (±SD)		5.7 (5.8)	6.3 (6.5)
	Week 12, mean (±SD)		6.2 (6.5)	5.6 (9.2)
Interleukin 6 (IL-6), pg/mL				
	Week 0, mean (±SD)		1.1 (0.6)	1.8 (1.3)
	Week 12, mean (±SD)		1.1 (0.5)	1.6 (1.3)
Interleukin 8 (IL-8), pg/mL				
	Week 0, mean (±SD)		22.7 (29.0)	35.0 (94.9)
	Week 12, mean (±SD)		20.3 (27.2)	20.7 (25.1)
Interleukin 10 (IL-10), pg/mL				
	Week 0, mean (±SD)		0.5 (0.5)	0.8 (0.94)
	Week 12, mean (±SD)		0.9 (1.7)	1.0 (2.5)
Tumor necrosis factor (TNF), pg/mL				
	Week 0, mean (±SD)		3.7 (5.1)	5.2 (10.9)
	Week 12, mean (±SD)		3.4 (2.7)	3.4 (3.7)

## Data Availability

The data underlying this article cannot be shared publicly due to the privacy of the individuals that participated in the study.
